# Ac2-26 Induces IKKβ Degradation Through Chaperone-Mediated Autophagy Via HSPB1 in NCM-Treated Microglia

**DOI:** 10.3389/fnmol.2018.00076

**Published:** 2018-03-15

**Authors:** Lu Liu, Dandan An, Junying Xu, Bin Shao, Xing Li, Jing Shi

**Affiliations:** ^1^Department of Neurobiology and Key Laboratory of Neurological Diseases of Ministry of Education, Tongji Medical College, Huazhong University of Science and Technology, Wuhan, China; ^2^Institute for Brain Research, Collaborative Innovation Center for Brain Science, Huazhong University of Science and Technology, Wuhan, China

**Keywords:** annexin-A1, OGD/R, HSP27, TNF-α, LAMP-2A, microglia

## Abstract

Annexin A1 (ANXA1) is an endogenous protein with potent anti-inflammatory properties in the brain. Although ANXA1 has been predominantly studied for its binding to formyl peptide receptors (FPRs) on plasma membranes, little is known regarding whether this protein has an anti-inflammatory effect in the cytosol. Here, we investigated the mechanism by which the ANXA1 peptide Ac2-26 decreases high TNF-α production and IKKβ activity, which was caused by oxygen glucose deprivation/reperfusion (OGD/R)-induced neuronal conditioned medium (NCM) in microglia. We found that exogenous Ac2-26 crosses into the cytoplasm of microglia and inhibits both gene expression and protein secretion of TNF-α. Ac2-26 also causes a decrease in IKKβ protein but not IKKβ mRNA, and this effect is inverted by lysosome inhibitor NH_4_CL. Furthermore, we demonstrate that Ac2-26 induces IKKβ accumulation in lysosomes and that lysosomal-associated membrane protein 2A (LAMP-2A), not LC-3, is enhanced in microglia exposed to Ac2-26. We hypothesize that Ac2-26 mediates IKKβ degradation in lysosomes through chaperone-mediated autophagy (CMA). Interestingly, ANXA1 in the cytoplasm does not interact with IKKβ but with HSPB1, and Ac2-26 promotes HSPB1 binding to IKKβ. Furthermore, both ANXA1 and HSPB1 can interact with Hsc70 and LAMP-2A, but IKKβ only associates with LAMP-2A. Downregulation of HSPB1 or LAMP-2A reverses the degradation of IKKβ induced by Ac2-26. Taken together, these findings define an essential role of exogenous Ac2-26 in microglia and demonstrate that Ac2-26 is associated with HSPB1 and promotes HSPB1 binding to IKKβ, which is degraded by CMA, thereby reducing TNF-α expression.

## Introduction

Worldwide, stroke is a leading cause of death and disability with limited therapeutic options (Macrez et al., 2011), and ischemic stroke alone represents 80%–85% of acute strokes (Flynn et al., 2008). Although different mechanisms underlie the pathogenesis of stroke, accumulating evidence has shown that inflammation accounts for its progression, particularly in acute cases (Chamorro and Hallenbeck, 2006). Among resident cells, microglia represent a probable primary source of inflammatory mediators in the CNS. Sudden occlusion of cerebral blood flow leads to energy depletion and necrotic neuronal death, which can trigger immune responses that ultimately lead to inflammatory cell activation. On one hand, lesioned neurons rapidly change their gene expression and generate multiple factors such as ATP and glutamate that stimulate nearby microglia activation and migration to protect the brain against ischemic and excitotoxic injury (Neumann, 2001; Lo et al., 2003; Luo et al., 2014). On the other hand, excessively activated microglia release a large variety of proinflammatory mediators, including reactive oxygen species (ROS), IL-1 and TNF-α, which exacerbate tissue damage and contribute to the delayed exacerbation of ischemic brain deficits (Nathan and Ding, 2010; Graeber et al., 2011; Fernandes et al., 2014; Tuttolomondo et al., 2014). Stroke and cerebral ischemic damage induce high serum and brain levels of TNF-α. Blocking endogenous TNF-α, which is mainly generated by microglia (Zhang et al., 2013), significantly reduces focal ischemic brain injury and infarct size (Barone et al., 1997).

Annexin A1 (ANXA1), an endogenous glucocorticoid mediator implicated in mediation of a broad range of anti-inflammatory actions (Yang et al., 2009; McArthur et al., 2010; Williams et al., 2010; Girol et al., 2013), inhibits NF-kB activation and proinflammatory cytokine production. In the brain, ANXA1 is abundant in microglial cells, which also express high levels of formyl peptide receptor (FPR; Young et al., 1999; Solito et al., 2008; Liu et al., 2016). Once released, ANXA1 or the N-terminal peptide Ac2-26 binds to pro-resolving FPRs to inhibit microglial activation and proinflammatory cytokine production (McArthur et al., 2010; Luo et al., 2014). However, in the assay described here, we found for the first time that exogenous Ac2-26 enters the cytosol of microglia without interacting with FPRs on the membrane. ANXA1 exerts many of its anti-inflammatory and pro-resolving effects through FPRs (Leoni et al., 2013), while the intracellular mechanisms involved in its cytosolactions have not been fully elucidated. In particular, the exact mechanism of exogenous ANXA1 in microglia affected by ischemic stroke remains unknown. The aim of the present study was to analyze the effects of Ac2-26 on TNF-α secreted from microglia following acute stroke and the underlying mechanism.

IKKβ/NF-κB signaling plays a critical role in microglial production of proinflammatory genes, including TNF-α, IL-1β and inducible nitric oxide synthase (iNOS; Frakes et al., 2014). Once activated, IκB kinase (IKK)-dependent phosphorylation and subsequent degradation of the specific inhibitor IκB leads to release of NF-κB and transcription of target genes (Ghosh and Karin, 2002). Deletion of IKKβ in the myeloid lineage reduces microglial activation and neuronal loss in models of excitotoxicity and ischemic brain injury (Cho et al., 2008). Our previous findings showed that ANXA1 could polarize microglia to the anti-inflammatory M2 phenotype to protect neurons from ischemia-like injury (Luo et al., 2014). Therefore, we deduced that the mechanism of ANXA1 in microglia may involve the IKKβ/NF-κB pathway.

Autophagy, a lysosomal degradation pathway, is involved in multiple cellular processes including the regulation and function of innate and adaptive immune responses (Levine et al., 2011; Valdor et al., 2014). Studies have shown that activation of autophagy may be involved in the mechanism of neuroprotection in the ischemic brain (Alirezaei et al., 2011; Galluzzi et al., 2016). According to the different pathways by which cytoplasmic materials are delivered to lysosomes or vacuoles, autophagy is divided into the following major types: macroautophagy, microautophagy and chaperone-mediated autophagy (CMA; Mizushima and Komatsu, 2011). In CMA, cytosolic substrates with a KFERQ-like motif are targeted by Heat shock-cognate protein of 70 Kda (Hsc70) and other chaperones that transport them to the lysosomal surface. After interacting with a lysosomal membrane receptor, lysosomal-associated membrane protein 2A (LAMP-2A), substrate proteins undergo unfolding and translocation across the membrane into the lysosomal lumen for degradation (Patel and Cuervo, 2015). In this process, LAMP-2A is considered the rate-limiting factor for target translocation into lysosomes. In the brain, LAMP-2A is mainly found in neurons and proliferating microglia (Park et al., 2015). Activation of CMA leads to robust neuroprotective effects (Dohi et al., 2012) and impairs autophagic function to varying degrees, thereby contributing to neurodegenerative diseases. However, no studies have explored the role of CMA in microglia after ischemic stroke.

In the present study, we investigated the mechanism of Ac2-26-attenuated TNF-α in microglia treated with oxygen glucose deprivation/reperfusion (OGD/R)-induced neuronal conditioned medium (NCM). We demonstrate that Ac2-26 provokes the association of IKKβ with the small molecular chaperone HSPB1, which leads to degradation of IKKβ by CMA. Inhibition of HSPB1 or LAMP-2A leads to excessive IKKβ activation and TNF-α secretion. Importantly, our data show that ANXA1, HSPB1 and IKKβ all bind to LAMP-2A and are degraded through CMA. Thus, for the first time, this study highlights the importance of the CMA pathway in microglia and provides a new mechanism by which exogenous Ac2-26 may be exploited as an anti-inflammatory therapy by inducing HSPB1 and promoting the degradation of IKKβ in microglia following stroke.

## Materials and Methods

### Animals

Sprague-Dawley rats (8–10 weeks old; body weight 250–270 g; male) were obtained from the Experiment Animal Center of Tongji Medical College, Huazhong University of Science and Technology. Rats were housed in temperature-controlled rooms and maintained on a 12-h/12-h light/dark cycle. All animal experiments were approved by Tongji Medical College Experimental Animal Ethics Committee (Wuhan, China). All procedures complied with the guidelines of the National Institutes of Health for the Care and Use of Laboratory animals (NIH Publication No. 8023, revised 1978).

### Primary Cell Culture

Primary neuronal cultures were obtained from embryos at embryonic d16–18 by a standard procedure as previously described (Li et al., 2016). Mechanically dissociated cells were placed in 6-well culture plates. Primary microglia cultures were prepared from 1- to 2-day-old neonatal SD rats. The cortices were gently dissociated, and cell suspensions were passed through a 70-μm pore filter. Cells were resuspended in high-glucose Dulbecco’s Modified Eagle’s Minimum Essential Medium (DMEM, ThermoFisher Scientific) supplemented with 15% fetal bovine serum (FBS, Australia Origin, ThermoFisher Scientific) and incubated in vented T75 flasks (Corning, BD) at 37°C. Primary microglia were collected from the cultures 10–14 d post-dissection by shaking at 400 rpm on a rotary shaker at 37°C for 6 h and plated on poly-*d*-lysine (Sigma)-coated plates in DMEM containing 10% FBS for attachment overnight.

### Oxygen Glucose Deprivation Reperfusion (OGD/R) and NCM Treatment

Oxygen glucose deprivation (OGD) was induced in primary neuronal or microglial cultures. Briefly, culture medium was replaced by glucose-free DMEM (ThermoFisher Scientific), and cells were placed in an oxygen-deprived (94%N_2_/5%CO_2_/1%O_2_) incubator at 37°C for 2 h. Control cells were incubated in high-glucose DMEM under normoxic conditions (95%O_2_/5%CO_2_) for the same time period. The cells were returned to normoxic conditions with regular medium to terminate OGD and begin reperfusion. NCM was collected 24 h later.

### Antibodies and Reagents

The primary antibodies used were as follows: rabbit monoclonal antibody (mAb) against IKKβ (#8943) and polyclonal antibodies against LC3(#12741), IKKβ (#2678), ANXA1 (#3299) and p62 (#5114) were obtained from Cell Signaling Technology. Goat polyclonal antibodies against HSPB1 (sc-1048) and TNFα (sc-1350); mouse polyclonal antibodies against Hsc70 (sc-7298), β-actin (sc-47778) and GADPH (sc-365042); and rabbit or mouse immunoglobulin G(IgG) were from Santa Cruz Biotechnology. Rabbit mAb against LAMP-2A (ab125068) and mouse mAb LAMP-1 (ab25630) were from Abcam. Rabbit polyclonal antibodies against phospho-IκBα were purchased from Ruiying Biotechnology (Wuhan, China). Alexa Fluor probe-labeled secondary antibodies were from Jackson ImmunoResearch Labs. The nFPR antagonist, N-tert-butoxy-carbonyl-methionyl-leucyl-phenylalanine (BOC-1), was obtained from MP Biomedicals. NH_4_CL and chloroquine were acquired from Sigma-Aldrich. Ac2-26 (Ac-AMVSEFLKQAWFIENEEQEYVQTVK) and FITC-Ac2-26 were synthesized by Bioyeargene Biotechnology (Wuhan, China).

### Adenovirus shRNAs and Plasmids

*LAMP-2A* shRNA (shLAMP-2A)-expressing adenovirus (target sequence: GACTGCAGTGCAGATGAAG; Massey et al., 2006), H*spB1* shRNA (shHSPB1)-expressing adenovirus (target sequence: GGAGATCACCATTCCGGTTAC) and scrambled sequence adenovirus were supplied commercially by Vigene Biosciences (Shandong, China). mRNA was knocked down by transfection of microglia cells with the indicated adenovirus. Infected cells were sorted by GFP expression. LAMP-2A and HSPB1 expression was assessed by immunoblot analysis with antibodies as indicated. pEGFP-hsp27 wt FL was a gift from Andrea Doseff (Addgene plasmid #17444); pcDNA-IKKβ-FLAG WT was a gift from Warner Greene (Addgene plasmid #23298); pEGFP-ANXA1 wt was kindly supplied by Li et al. (2016); pEGFP-ANXA1-N and Flag-Ac2-26 were constructed with standard procedures; HA-LAMP-2A WT was purchased from Vigene Biosciences (Shandong, China). The Hsc70 shRNA plasmid (sc-29349-SH) was purchased from Santa Cruz Biotechnology.

### Cell Culture and Transfection

BV-2 and HeLa cells were cultured in high-glucose DMEM supplemented with 10% (v/v) FBS (ThermoFisher Scientific) and incubated at 37°C under humidified atmosphere of 5% (v/v) CO_2._ Transfection of BV2 cells was carried out using Lipofectamine 2000 (ThermoFisher Scientific), as recommended by the manufacturer. At 48 h after transfection with various plasmids, the cells were subjected to Western blotting analysis. For Ac2-26 treatment, the cells were treated with 10 μM Ac2-26 for 24 h before harvesting (Hayhoe et al., 2006 #1028). In some experiments, microglia cells were pretreated with BOC-1 (5 μM) for 0.5 h (Luo et al., 2014 #187) or lysosomal protease inhibitor NH_4_CL (5 mM) for 2 h (Andersson et al., 2005 #1027).

### Lysosome Isolation

Lysosomes were isolated from brain tissue using a Lysosome Isolation kit (Sigma) according to the kit protocol. Briefly, 4 g rat brain tissue was homogenized in 16 ml ice-cold extraction buffer containing 1× protein inhibitor cocktail at 8000 rpm for 5 s, followed by 9500 rpm for two additional 5-s periods. The homogenate was centrifuged at 1000× *g* for 10 min at 4°C. The obtained supernatant (4 ml) was then centrifuged at 20,000× *g* for 20 min at 4°C. The supernatant was diluted in a solution containing 19% OptiPrep density gradient medium. The mixed medium was separated by density gradient centrifugation (150,000× *g* for 4 h) with Beckman Coulter Optimal L-80 XP Ultracentrifue and SW 55Ti Swing out Rotor in a multistep OptiPrep gradient, and calcium chloride was added to a final concentration of 8 mM for low-speed (5000× *g* for 15 min) centrifugation. The top 3 ml was collected as the lysosomal fraction and stored at 4°C until use for Western blotting. Lysosomal integrity was assessed using Neutral Red dye (Sigma).

### Western Blot

Cells were lysed in RIPA buffer (Beyotime Biotechnology, Shanghai, China) supplemented with a protease inhibitor mixture. Protein samples (60 μg) were heated at 98°C for 10 min and separated on 10% or 12% SDS-PAGE gels. The proteins were then transferred to a polyvinylidene fluoride membrane (PVDF; Roche). The membranes were incubated with 5% fat-free milk in TBST for 1 h and probed with the antibody (Ab) of interest overnight at 4°C, followed by HRP-conjugated secondary Ab and developed by chemiluminescence detection (ECL; Advance) with autoradiography (Bio-Rad). Band densitometry was analyzed by Image Lab^TM^ Software, Version 5.1 (Bio-Rad). All Western blot data are expressed as the ratio of the levels of the protein of interest and β-actin.

### Co-immunoprecipitation

Cell lysates were centrifuged (12,000× *g*) at 4°C for 15 min. Proteins were immunoprecipitated with indicated antibodies and shaken at 4°C overnight. Then, 40 μL precleared Protein A/G Plus-agarose beads (Beyotime Biotechnology, Shanghai, China,) was incubated with immunocomplexes for 3 h at 4°C and centrifuged at 1000× *g* for 5 min. The supernatant was discarded, and the pellet was washed 5 times with PBS. Next, the precipitates were resuspended with 1× sample buffer and heated at 98°C for 5 min. The samples were centrifuged for 5 s, and the supernatants were collected for Western blotting. Immunoblotting analysis was performed as previously described.

### RT-PCR and Quantitative Real-Time PCR

Total RNA from microglia was extracted by TRIzol and converted into cDNA using a Revertra Ace qPCR RT Kit (both from Toyobo) according to the manufacturer’s protocols. Real-time PCR was performed using SYBR Green I PCR Master Mix in a StepOnePlus Real-Time PCR System (Applied Biosystems). The data were analyzed using StepOne System software with a cycle threshold (Ct) in the linear range of amplification and then processed by the 2^−ΔΔCt^ method.

The following primers were used:

- *TNF-α*: forward, 5′- CGTGTTCATCCGTTCTCTACC-3′;
- *TNF-α*: reverse, 5′- CGTGTGTTTCTGAGCATCGT-3′;
- *IKKβ*: forward, 5′- TTGTGGGGACCCTGCAATAC-3′;
- *IKKβ*: reverse, 5′- GCCGAAGCTCCAGTAGTCAA-3′;
- *β-actin*: forward, 5′- CACCCGCGAGTACAACCTTC-3′;
- *β-actin*: reverse, 5′- CCCATACCCACCATCACACC-3′.

All data were reinterpreted as fold changes relative to untreated control samples.

### ELISA

Microglia were seeded in PDL-coated 24-well plates at 3 × 10^4^ cells/well, and TNF-α levels were determined by a rat TNF-α ELISA kit (BioLegend) according to the kit protocol.

### Immunofluorescence and Confocal Microscopy

Primary microglia were collected and washed with PBS 3 × 5 min, followed by fixation with 4% paraformaldehyde (PFA) at room temperature for 15 min and permeabilization with 0.1% Triton X-100 for 10 min. Cells were then blocked in 10% goat serum (ThermoFisher Scientific) in PBS for 1 h at 22°C, followed by staining with a mAb for LAMP-2A (1:200, Abcam) and LAMP-1 (1:200, Abcam) at 4°C overnight. Cultures were subsequently washed three times in PBS and counterstained with Alexa Fluor 488 goat anti-rabbit IgG or Alexa Fluor 594 goat anti-mouse IgG for 1 h at room temperature. The cells were subsequently washed three times in PBS and stained with DAPI (Abcam) for 5 min at room temperature. Finally, the cultures were analyzed with a confocal microscope (LEICA TCS SP5II, Germany). Images were digitally analyzed by Image-Pro Plus software to quantify the fluorescence intensity of cells.

### Statistical Analysis

All experiments were performed a minimum of three times (independently) and are expressed as the mean values ± SEM. Statistically significant differences between groups were identified by one-way analysis of variance (ANOVA) with Tukey’s multiple comparison tests or unpaired Student’s *t*-test. *P* < 0.05 was considered statistically significant. The statistical software used was Prism v.6 (GraphPad Software).

## Results

### Ac2-26 Inhibits NCM-Induced TNF-α Production in Microglia

**Figure 1:**
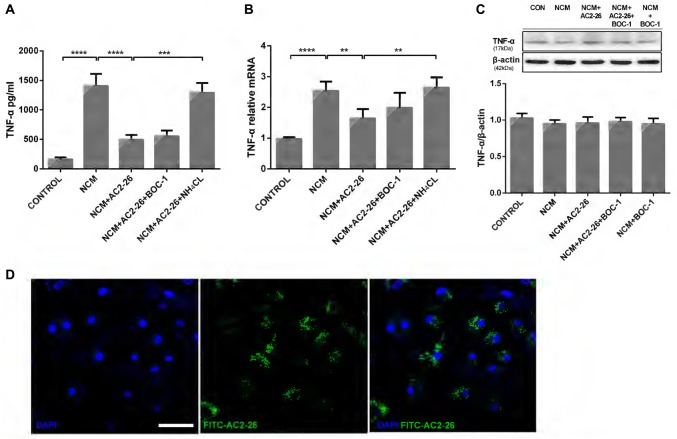
Ac2-26 promotes the inhibition of TNF-α production in microglia following neuronalconditioned medium (NCM). Microglia were cultured for 24 h in NCM stimulated with Ac2-26 (10 μM) in the presence or absence of BOC-1 (5 μM) and NH_4_CL (5 mM) as indicated. The supernatants were collected for cytokine analysis by ELISA **(A)**, and cell pellets were collected for real-time quantitative PCR **(B)** or Western blotting **(C)**. The data are representative of 3–4 independent experiments and were analyzed by one-way analysis of variance (ANOVA) with Tukey’s multiple comparison tests. Throughout the figure, the error bars represent the mean ± SEM. **P < 0.01; ***P < 0.001; ****P < 0.0001 vs. control. **(D)** Microglia were incubated with the FITC-Ac2-26 peptide for 24 h and detected with confocal microscopy. The bar represents 50 μm.

Ischemic brain injury resulting from stroke arises from primary neuronal losses and inflammatory responses. Neuronal cell death leads to the transformation of microglia into phagocytotic cells and their migration to lesion sites to remove cellular debris (Kettenmann et al., 2013), which in turn injures otherwise viable cells (Wang et al., 2007; Yenari et al., 2010). To better simulate the internal environment in the brain after stroke, we applied NCM for 24 h to trigger hypoxia-ischemia in microglia and detected high levels of TNF-α in the supernatant. Administration of Ac2-26 reduced NCM-induced TNF-α release, which was reversed by the lysosomal inhibitor NH_4_CL but not the FPR receptor antagonist BOC-1 (Figure 1A). Consistent with this finding, microglia cultured in NCM for 24 h exhibited a strong increase in TNF-α mRNA expression, which was reversed by Ac2-26 treatment and induced in the presence of NH_4_CL but not BOC-1 (Figure 1B). Note that concomitant intracellular protein expression of TNF-α was not significantly changed after the different treatments (Figure 1C), but total protein, including secretion of TNF-α, decreased after Ac2-26. Therefore, we hypothesized that Ac2-26 affects TNF-α secretion and gene expression in microglia and that reduced TNF-α is associated with autophagy. Regarding the failure of BOC-1 to induce TNF-α, we contemplated whether Ac2-26 reduced TNF-α production in the cytosol of microglia without binding to FPRs on the membrane. To test this hypothesis, exogenous FITC-Ac2-26 was cultured with microglia. Confocal microscopy experiments with microglia revealed that most peptides were distributed diffusely through the cytoplasm but not in the membrane or nuclei (Figure 1D). These data indicated that exogenous Ac2-26 serves anti-inflammatory functions in the cytoplasm of microglia.

### Ac2-26 Decreases NCM-Induced IKKβ Activity Through Autophagy

**Figure 2:**
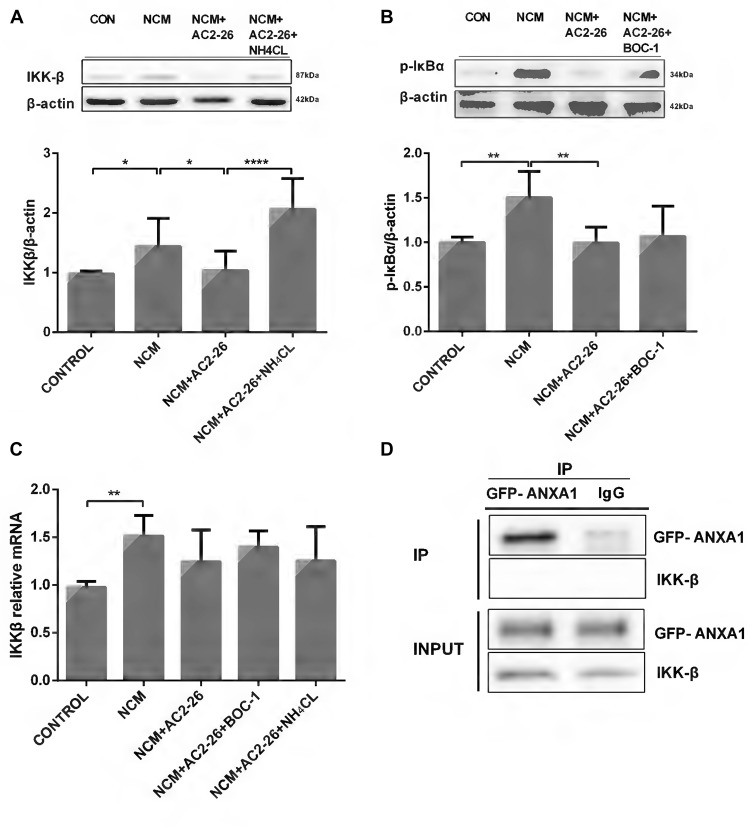
IKKβ activity is suppressed by Ac2-26 in NCM-treated microglia.** (A)** Western blot analysis showing IKKβ expression in microglial cells following 24 h of NCM in the presence or absence of Ac2-26 (10 μM) or NH_4_CL (5 mM). **(B)** Western blot analysis showing phospho-IκBα expression in microglia pretreated with or without BOC-1 (5 μM) and incubated with NCM or Ac2-26 (10 μM) for 24 h. **(C)** The mRNA expression levels of IKKβ were determined by real-time quantitative PCR and normalized to β-actin. Cells were treated as indicated. The data are representative of 4–6 independent experiments. Statistical significance was assessed using one-way ANOVA with Tukey’s multiple comparison tests. Throughout the figure, the error bars represent the mean ± SEM. *P < 0.05; **P < 0.01; ****P < 0.0001 vs. control. **(D)** Protein cell lysates from HeLa cells transfected with GFP-ANXA1 were immunoprecipitated with anti-GFP monoclonal antibody (mAb) or mouse IgG and immunoblotted using an anti-IKKβ mAb. This experiment was repeated at least three times.

Next, we questioned the role of Ac2-26 in microglia and how it inhibits TNF-α expression. The canonical IKKβ/NF-κB activation pathway involved in regulation of inflammation is the major upstream pathway of TNF-α (Maeda et al., 2003; Yang et al., 2003). Therefore, we sought to determine whether the Ac2-26-induced decline in TNF-α was dependent on the IKKβ/NF-κB signaling pathway. As shown in Figure 2A, NCM-induced IKKβ protein expression was inhibited by Ac2-26 in microglia. Because IKKβ is the kinase responsible for IκBα phosphorylation, which leads to IκBα degradation and NF-κB activation, we measured phospho-IκBα levels in microglia. The results showed the same patterns as IKKβ activity (Figure 2B), indicating that Ac2-26 inhibits TNF-α production through an IKKβ-dependent pathway. To further delineate IKKβ expression, we assessed IKKβ mRNA levels. No significant differences were found after Ac2-26 treatment despite the elevation observed for NCM-only treatment (Figure 2C), suggesting that the reduction in IKKβ protein is due to an increase in degradation. Furthermore, BOC-1 revealed no changes in phospho-IκBα protein or IKKβ mRNA (Figures 2B,C), confirming that FPR does not participate in the role of Ac2-26. In addition, NH_4_CL clearly reversed the Ac2-26-induced IKKβ decrease without affecting its mRNA (Figures 2A,C), indicating that post-translational modifications for lysosomal degradation may be involved in the mechanism of Ac2-26-induced IKKβ reduction. Given these data, we hypothesized that more IKKβ protein would be produced when microglia were stimulated with NCM and degrade faster with a steady rate of synthesis if Ac2-26 was applied. Moreover, co-immunoprecipitation (CO-IP) analysis revealed that ANXA1 had no marked interaction with IKKβ (Figure 2D), which indicated that Ac2-26 does not directly play a role in IKKβ degradation or that the interaction is too weak to detect.

### Ac2-26 Induces HSPB1 Expression in Microglia

**Figure 3:**
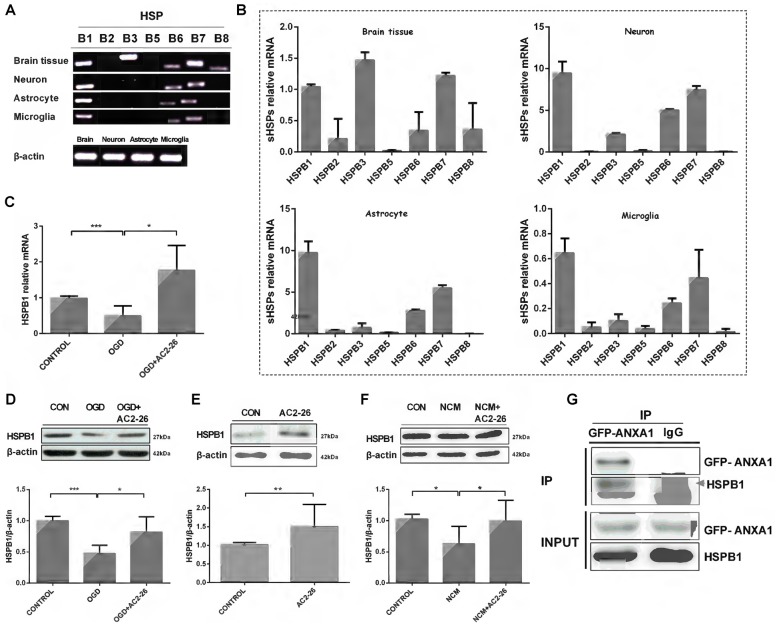
HSPB1 is induced by Ac2-26 in microglia. RT-PCR analysis of total RNA isolated from rat brain tissues, neurons, astrocytes and microglial cells revealed the presence of sHSP transcripts **(A,B)**. BV-2 cells were treated with Ac2-26 (10 μM) before they were continuously exposed to oxygen glucose deprivation (OGD) for 1 h and reperfusion for 24 h. The mRNA expression of HSPB1 was assessed by RT-PCR **(C)**, and the protein levels were detected by Western blotting **(D)** as indicated. **(E,F)** Microglia were treated with NCM or Ac2-26 (10 μM) for 24 h. Protein levels were assessed by Western blotting as indicated. The data are representative of at least three independent experiments and shown as the mean ± SEM. All data were analyzed using one-way ANOVA with Tukey’s multiple comparison tests; Ac2-26 induced HSPB1 protein expression **(E)** was analyzed using unpaired Student’s *t* test. *P < 0.05; **P < 0.01; ***P < 0.001 vs. control. **(G)** Protein cell lysates from HeLa cells transfected with GFP-ANXA1 were immunoprecipitated with anti-GFP mAb or mouse IgG and immunoblotted using an anti-HSPB1 mAb. This experiment was repeated at least three times.

Next, we sought to determine the factors that assist in IKKβ degradation. Small heat shock proteins (sHSPs) are a family of molecular chaperones that may exhibit immunomodulatory and anti-inflammatory functions (Shao et al., 2013; Bakthisaran et al., 2015). To verify a series of sHSPs expressed in the brain, we evaluated whole brain tissue, neurons, astrocytes and microglia with PCR (Figures 3A,B). HSPB1, also known as HSP27, exhibited high levels in neurons, astrocytes and microglia. HSPB1 induces beneficial outcomes in neuroprotection, and overexpression of HSPB1 in transgenic animals confers robust cellular protection against a variety of neurological insults and diseases, including cerebral and cardiac ischemia (Stetler et al., 2009; van der Weerd et al., 2010). Thus, we first examined the involvement of HSPB1 in Ac2-26 treated microglia. We used OGD/R to directly trigger hypoxia-ischemia in microglia *in vitro*. In contrast to neuronal cells in which the expression of HSPB1 is selectively induced under stress (Quraishe et al., 2008), both protein and mRNA expression levels of HSPB1 in microglia were inhibited after OGD and reversed by Ac2-26 (Figures 3C,D). Furthermore, HSPB1 increased in primary microglia treated with Ac2-26 alone or before NCM (Figures 3E,F). According to the above results, both Ac2-26 and HSPB1 exist in the cytoplasm, and Ac2-26 promotes HSPB1 expression. We also sought to determine if Ac2-26 directly targets HSPB1 in the cytosol; Figure 3G reveals interaction of ANXA1 and HSPB1. To further analyze the domain of ANXA1 binding to HSPB1, we constructed a plasmid with 1–43 amino acid residues from the N-terminal of ANXA1 (Rosengarth et al., 2001 #1029) with GFP tag (GFP-ANXA1-N), and found HSPB1 only in GFP-ANXA1-N expressed cells (Supplementary Figure S1A). Further, we transferred the plasmid Flag-Ac2-26 into cells. Co-immunoprecipitation result showed that HSPB1 interacted with Ac2-26 (Supplementary Figure S1B). Together, these results indicated that Ac2-26 physically associates with HSPB1 and induces its expression.

### HSPB1 Negatively Regulates IKKβ Activation

**Figure 4:**
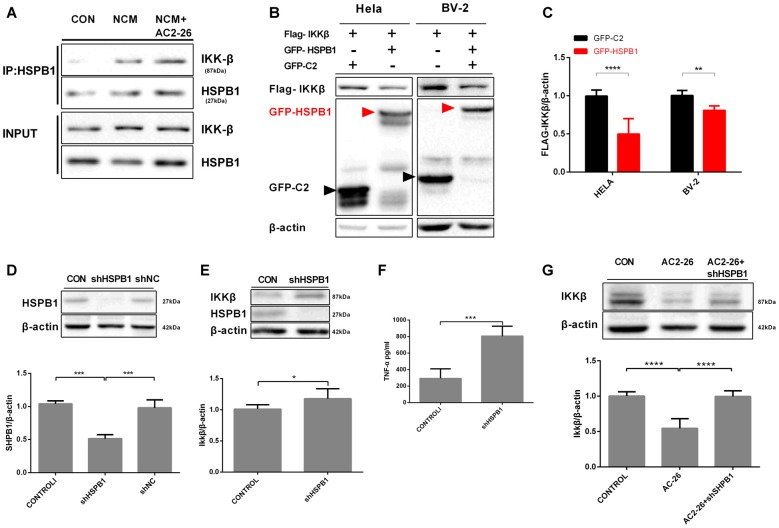
Interaction between HSPB1 and IKKβ. **(A)** The interaction between HSPB1 and IKKβ in microglia was assayed by CO-IP. Microglia were incubated with NCM in the presence or absence of Ac2-26 (10 μM) for 24 h. Cell lysates were immunoprecipitated with anti-HSPB1 and immunoblotted with anti-IKKβ. **(B)** HSPB1 overexpression reduced IKKβ levels. HeLa or BV-2 cells were transfected with vectors expressing GFP-tagged HSPB1 (7.5 μg/well) or FLAG-tagged IKKβ (7.5 μg/well) as indicated. Whole cell lysates were detected with anti-FLAG and anti-GFP. **(C)** Fold change of the IKKβ/β-actin ratio in the indicated conditions. **(D)** The expression of HSPB1 was knocked down in microglia using adenovirus carrying HSPB1-specific shRNA as described in the “Materials and Methods” section but not in cells transfected with control shNC. **(E,F)** Microglia were transfected with adenovirus carrying shRNA against HSPB1. At 48 h post-transfection, IKKβ protein levels were detected by immunoblotting **(E)**, and TNF-α production was assessed by ELISA **(F)**. Microglial cells transfected with shHSPB1 were then treated with Ac2-26 (10 μM), and protein levels were measured by Western blotting **(G)** as indicated. The data are representative of 3–7 independent experiments. Throughout the figure, the error bars represent the mean ± SEM. Data were analyzed using one-way ANOVA with Tukey’s multiple comparison tests between three groups and unpaired Student’s *t* test with two groups. *P < 0.05; **P < 0.01; ***P < 0.001; ****P < 0.0001 vs. control.

Regarding IKKβ degradation, we explored whether the high level of HSPB1 triggered by Ac2-26 may assist IKKβ as a molecular chaperone for autophagy. The above data showed that both IKKβ reduction and HSPB1 elevation were induced by Ac2-26 in microglia; these results suggested a possible interaction between HSPB1 and IKKβ. First, we examined the association in NCM-treated microglia with or without Ac2-26. Immunoprecipitation experiments demonstrated that HSPB1 was slightly associated with IKKβ under resting physiological conditions in microglia (Figure 4A), which is consistent with previous reports in HeLa cells by other groups (Park et al., 2003). In addition, NCM stimulation encouraged HSPB1 to associate with IKKβ in microglia, and Ac2-26 further enhanced this interaction (Figure 4A). These results indicated that IKKβ degradation may be correlated with HSPB1 induction. We next explored the interdependence between IKKβ and HSPB1 in knockdown and overexpression experiments. As shown in Figures 4B,C, overexpression of GFP-HSPB1 reduced FLAG-IKKβ in both HeLa and BV-2 cells. To examine the impact of HSPB1 loss, we downregulated HSPB1 in primary microglia by adenoviral vectors expressing specific shRNA (Figure 4D) and detected marked differences in IKKβ and TNF-α expression between treated microglia and control microglia (Figures 4E,F). Given these data, it is plausible that HSPB1 may serve as a negative regulator of IKKβ to eliminate NCM-triggered inflammation. Knockdown of HSPB1 restored the loss of IKKβ induced by Ac2-26 (Figure 4G), supporting the idea that HSPB1 is required for Ac2-26-induced IKKβ degradation.

### Ac2-26 Promotes CMA in Microglia

**Figure 5:**
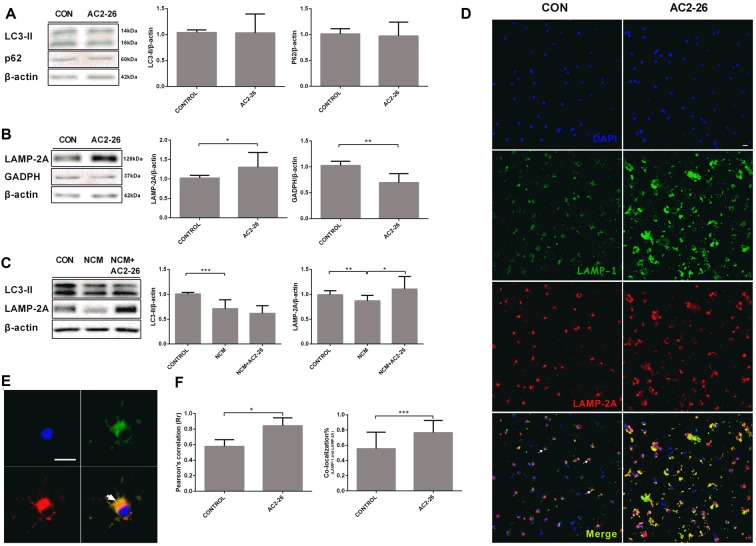
Chaperone-mediated autophagy (CMA) is activated in microglia exposed to Ac2-26.** (A)** Representative immunoblots ofLC3-II and P62 in microglia treated with Ac2-26 (10 μM; left); quantitative analysis of the levels of LC3-II and P62 (right). **(B)** Representative immunoblots of LAMP-2A and GADPH in microglia treated with Ac2-26 (10 μM; left); quantitative analysis of the levels of LAMP-2A and GADPH (right). **(C)** Representative immunoblots of LC3-II and LAMP-2A in microglia treated with Ac2-26 (10 μM) in the presence of NCM as indicated (left); quantitative analysis of the levels of LC3-II and LAMP-2A (right). The amount of each protein was normalized against that of β-actin. **(D,E)** Immunofluorescence for LAMP-1 and LAMP-2A in rat microglia treated with or without Ac2-26 (10 μM). The scale bar represents 20 μm. **(F)** Colocalization staining area with respect to the total cellular area (left). Pearson’s coefficient (r) indicates the correlation of the intensity values of the red and green pixels in the dual-channel images (right). The arrowheads indicate colocalization. The data are representative of at least three independent experiments and shown as the mean ± SEM. *P < 0.05; **P < 0.01; ***P < 0.001 vs. control.

An important question regarding autophagy related to IKKβ reduction is whether an autophagic pathway is responsible for Ac2-26-mediated inflammation. We monitored the shift of LC3-I to LC3-II, a canonical autophagosome marker. Primary microglia were cocultured with Ac2-26 for 24 h, and the results showed no changes in the conversion to LC3-II and accumulation of p62 (Figure 5A), which is considered a substrate of the autophagy process. In contrast, Ac2-26 enhanced CMA activation, as shown by significantly increased LAMP-2A accompanied by reduced GADPH, which is a CMA substrate protein (Figure 5B), suggesting that CMA, not macroautophagy, was induced by Ac2-26 in microglia. Accumulating evidence has shown that CMA is activated in brain tissues and neuronal cells after ischemic stimulation (Dohi et al., 2012). However, both LC3-II and LAMP-2A were decreased in microglia in response to NCM exposure, and only LAMP-2A expression was restored by Ac2-26 (Figure 5C). To further confirm that the increased LAMP-2A was mainly located in lysosomes, lysosomal marker LAMP-1 was used for colocalization with LAMP-2A in confocal immunofluorescence (Figures 5D,E). Ac2-26-treated microglia showed 19% more colocalization than did control microglia (Figure 5F). Thus, these results indicated that Ac2-26 specifically upregulated the expression of LAMP-2A in lysosomes and promoted CMA activity in microglia.

### ANXA1 and HSPB1 Assist the Translocation of IKKβ Into Lysosomes

**Figure 6:**
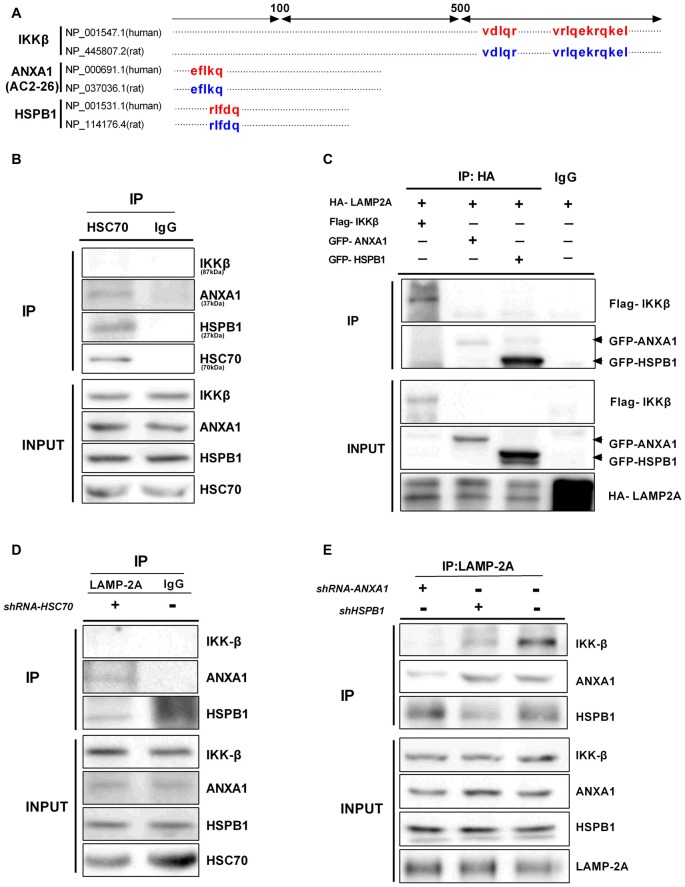
ANXA1 and HSPB1 transport IKKβ into lysosomes.** (A)** Amino acid locations of the putative CMA-targeting motifs in IKKβ, ANXA1 (Ac2-26) and HSPB1 proteins. **(B)** HeLa cells were immunoprecipitated with anti-Hsc70 and immunoblotted with anti- IKKβ, ANXA1, HSPB1 and Hsc70 antibodies. **(C)** LAMP-2A interacts with IKKβ, ANXA1 and HSPB1. HeLa cells were transfected with the indicated plasmids (100 μg each per 100-mm dish). The interaction was assessed by IP with anti-HA, followed by IP of Flag or GFP as indicated. **(D)** Silencing Hsc70 disrupted the binding of LAMP-2A and IKKβ. Anti-LAMP-2A immunoprecipitates from HeLa cells transfected with control or Hsc70 shRNA were immunoblotted with anti-IKKβ, ANXA1 and HSPB1 antibodies. **(E)** Anti-LAMP-2A immunoprecipitates from HeLa cells were transfected with control ANXA1 shRNA or adenovirus carrying HSPB1-specific shRNA and immunoblotted with anti-IKKβ, ANXA1 and HSPB1 antibodies. All data are representative of 3–4 independent experiments.

Proteins undergoing CMA-mediated lysosomal degradation often contain a loosely defined KFERQ motif recognized by Hsc70 and bind to the cytosolic tail of LAMP-2A (Kaushik et al., 2011). The KFERQ motif usually contains five residues, including a critical glutamine (Q) residue, which precedes four amino acids consisting of a basic residue (R or K), an acidic residue (E or D), or a bulky hydrophobic residue (I, L, V, or F; Dice, 2007). We noted that IKKβ, ANXA1 (Ac2-26) and HSPB1 all bear putative CMA motifs in their amino acid sequence (Figure 6A), but these motifs do not indicate that the proteins are continuously degraded through CMA (Klionsky et al., 2016). In CMA, Hsc70 functions to recruit target proteins to the lysosome for degradation. The endogenous interaction between Hsc70 and ANXA1 was confirmed by CO-IP (Figure 6B), which is in agreement with a previous study by other groups in which ANXA1 was selectively degraded by CMA (Cuervo et al., 2000). Similarly, we found an association between endogenous Hsc70 and HSPB1 but not IKKβ (Figure 6B). These results indicate that IKKβ may not be a substrate for the classical CMA pathway. LAMP-2A is a limiting factor of CMA and acts as a receptor for CMA substrates. To further confirm that these proteins are degraded by CMA, we co-transfected HeLa cells with a HA-tagged form of LAMP-2A and Flag-IKKβ, GFP-ANXA1 or GFP-HSPB1 plasmids. Surprisingly, all three proteins, including IKKβ, precipitated with HA-LAMP-2A (Figure 6C). Moreover, endogenous IKKβ, ANXA1 and HSPB1 associated with LAMP-2A (Figure 6E), which corroborated previous results. Given the interaction among these protein complexes, we contemplated whether IKKβ was carried by ANXA1 or HSPB1 for introduction into lysosomes. To test this hypothesis, cells were transfected with Hsc70 shRNA and immunoprecipitated with a LAMP-2A-specific Ab. IKKβ was almost undetectable regardless of the faint expression of ANXA1 and HSPB1. These data indicated that degradation of IKKβ is Hsc70 independent (Figure 6D). Compared with control, knockdown of ANXA1 completely blocked the interaction between LAMP-2A and IKKβ, while suppression ofHSPB1 resulted in weaker binding (Figure 6E). Together, these results suggested that ANXA1 and HSPB1 assist in the translocation of IKKβ into lysosomes.

### Degradation of IKKβ in Lysosomes by CMA

**Figure 7:**
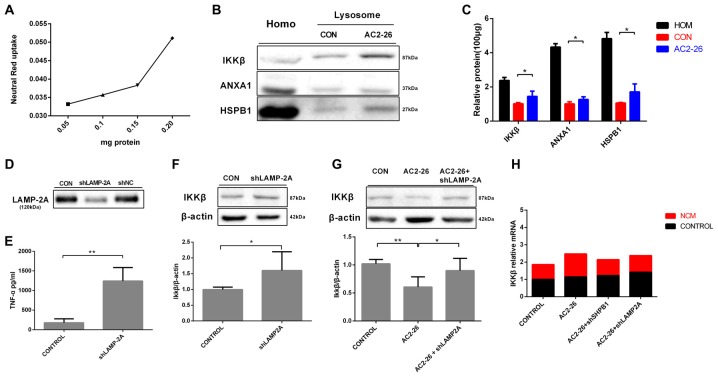
IKKβ is degraded by CMA.** (A)** Linearity of dye uptake with increasing amounts of lysosomal protein. **(B)** Homogenates (Homo) and lysosomes isolated from rat brain tissues were pretreated with chloroquine (50 μM) for 30 min at 4°C prior to the addition of Ac2-26(1 mg/g) for an additional 30 min. A total of 100 μg of protein was then processed and analyzed by Western blotting as indicated. **(C)** The protein levels of IKKβ, ANXA1, and HSPB1 per 100 μg of total protein from rat brain tissues. **(D)** Microglia were transfected with adenovirus carrying shRNA against LAMP-2A. LAMP-2A levels were examined by Western blotting. **(E,F)** Microglial cells were transfected with LAMP-2A shRNA. The supernatants were assessed for TNF-α by ELISA **(E)**, and the cell lysates were analyzed with antibodies to IKKβ and β-actin by Western blotting **(F)**. Microglial cells transfected with shLAMP-2A were then treated with Ac2-26, and protein levels were measured by Western blotting **(G)** as indicated. The data are representative of 3–6 independent experiments and were analyzed by one-way ANOVA with Tukey’s multiple comparison tests between three groups and unpaired Student’s *t* test between two groups. Throughout the figure, the error bars represent the mean ± SEM. *P < 0.05; **P < 0.01 vs. control. **(H)** Microglia transfected with shHSPB1 or shLAMP-2A for 48 h in the presence or absence of NCM for 24 h. The expression levels of IKKβ mRNA were determined by real-time quantitative PCR and normalized to β-actin. The levels of untreated control cells (CON) were set to100%. Data were analyzed by one-way ANOVA with Tukey’s multiple comparison tests.

**Figure 8:**
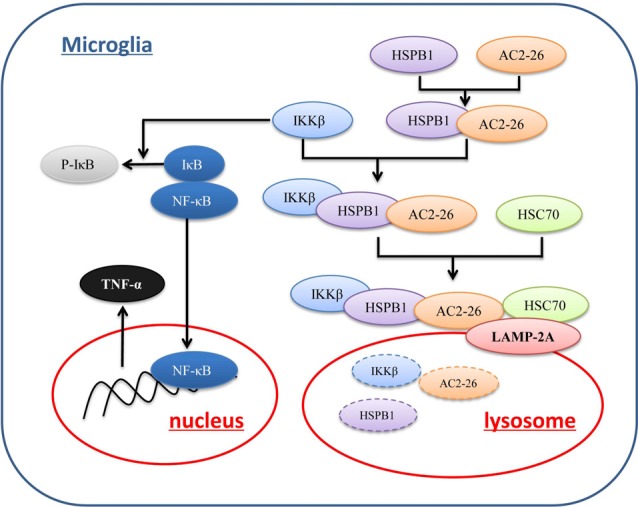
Proposed mechanism of Ac2-26-induced CMA-dependent degradation of IKKβ. Ac2-26 enters microglial cells to bind to and induce HSPB1, and HSPB1 further associates with IKKβ. The Ac2-26-HSPB1-IKKβ protein complex is recruited by a chaperone complex containing Hsc70 and binds to LAMP-2A on lysosomes. Ac2-26 and HSPB1 then assist with the translocation of IKKβ through LAMP-2A multimers into lysosomes for degradation.

To further evaluate the degradation of these proteins by potential uptake in lysosomes, we assessed the accumulation of IKKβ, ANXA1 and HSPB1 in lysosomes from rat brains by immunoblotting. We pretreated brain tissue with chloroquine, a lysosomal inhibitor, prior to treatment with Ac2-26 as any lysosome-internalized substrate is rapidly degraded by the luminal protease. The integrity of the lysosomes was assessed by Neutral Red dye (Figure 7A). As shown in Figure 7B, all three proteins (IKKβ, HSPB1 and endogenous ANXA1) were detected to some extent in the isolated lysosomes. In addition, the amount of IKKβ, HSPB1 and endogenous ANXA1 in lysosomes was higher in Ac2-26-treated tissue than in control tissue (Figure 7C). These findings suggested that Ac2-26 promotes both IKKβ and HSPB1 transport into lysosomes for further degradation. A critical piece of evidence for CMA involvement in downregulation of substrates was obtained by turning off LAMP-2A in cells and assessing the intracellular levels of the candidate substrates. To evaluate the contribution of CMA to IKKβ degradation, we knocked down LAMP-2A in microglia by adenovirus transfection of a shRNA against LAMP-2A. Immunoblotting results confirmed that LAMP-2A levels were successfully knocked down in microglia (Figure 7D). Strikingly, depletion of LAMP-2A, the rate-limiting factor in the CMA process, resulted in further accumulation of IKKβ (Figure 7F) and subsequent TNF-α secretion (Figure 7E), indicating that IKKβ is a CMA substrate. In addition, compared with Ac2-26 administration alone, knockdown of LAMP-2A before Ac2-26 administration dramatically reduced the degradation of IKKβ (Figure 7G). These data implied that CMA is required for the rapid degradation of IKKβ. Consistently, knockdown of neither HSPB1 nor LAMP-2A affected IKKβ mRNA (Figure 7H). Hence, we propose that Ac2-26 coupled with IKKβ targets LAMP-2A for lysosomal degradation (Figure 8).

## Discussion

Microglia are the major resident immune cells involved in the defense against brain damage. When microglia are excessively activated by damage-associated molecular patterns following stroke, they can produce many proinflammatory cytokines that can disrupt neural cells and influence neurogenesis. We have reported the importance of ANXA1 in driving microglial activation and migration to protect neurons from ischemia-like injury (Luo et al., 2014; Liu et al., 2016). In this study, exposure of microglia to NCM induced elevated expression of TNF-α, which was downregulated by Ac2-26. However, compared with primary microglia, BV-2 and HAPI cells (microglial cell lines) showed different patterns in experiments assessing TNF-α or IL-1β release (data not shown). Horvath et al. (2008) also suggested that BV-2 and HAPI cell cultures only partially model primary microglia and should be used with caution.

Endogenous ANXA1 is associated with the treatment of unresolved inflammation (Vago et al., 2016), and overexpression of ANXA1 resolves neutrophilic inflammation induced by LPS in mice (Vago et al., 2015). However, in breast cancer cells, cytoplasmic ANXA1 can activate NF-κB either directly by interacting with and stabilizing the NEMO-RIPI-IKK complex (Bist et al., 2011) or indirectly by inhibiting miR-26b and miR-562, which directly silence the NF-κB pathway by recognizing p65 and p105 mRNAs at the 3′-UTR (Anbalagan et al., 2014). We reasoned that this conflict may be due to differences between normal and tumor cell lines. Thus, we proposed that ANXA1 contributes to the resolution of inflammation from non-tumor cells but otherwise exerts opposite effects in tumor cells (Bist et al., 2015). This hypothesis also supports the differences in cytokines between primary microglial and glioma cell lines. Therefore, we conducted most experiments in primary microglia.

We observed that exogenous Ac2-26 was mainly located in the cytosol of microglia. The primary mechanism by which ANXA1 is known to mediate its effects is through FPRs. Exogenous ANXA1 functions through FPRs to initiate endogenous pro-resolving and anti-inflammatory pathways following ischemic stroke (Vital et al., 2016), but the roles of its peptides in microglia remain unclear. In many instances, the protein is externalized from the cell during cell activation. ANXA1 can be externalized through several mechanisms involving direct interaction with the plasma membrane, membrane transporters or vesicular trafficking, depending on the cell type (Boudhraa et al., 2016). To date, FPRs are the only known receptors of externalized ANXA1. All these results directed us to focus on the effect of ANXA1 on FPRs, although the mechanisms of ANXA1 that remains in the cytoplasm or exogenous Ac2-26 in the cytosol are seldom reported. We hypothesized that ANXA1 acts in concert with pro-resolving molecules in microglia. In the present work, we demonstrated for the first time that ANXA1 associates with HSPB1 in the cytosol and elevates its expression combined with downregulated IKKβ activity.

The small molecular weight heat shock protein, HSPB1, is upregulated in both neurons and astrocytes of different animal species under stress and neurodegenerative conditions (Filipcik et al., 2015; Imahori et al., 2017). In microglia, the role of HSPB1 and the underlying molecular mechanisms have not yet been reported. In this study, for the first time, we noticed that HSPB1 was significantly decreased in microglia subjected to OGD/R and upregulated after Ac2-26 treatment. HSPB1 exerts powerful neuroprotective effects, and overexpression of HSPB1 in transgenic animals confers robust cellular protection against a variety of neurological insults and diseases, including cerebral ischemia (van der Weerd et al., 2010; Bakthisaran et al., 2015). The molecular mechanisms underlying HSPB1 neuroprotection in specific types of cells remain under investigation. To our knowledge, this study is the first to identify HSPB1 as a negative regulator of IKKβ in microglia, which phosphorylates IκBα, leading to NF-κB activation. This result is in agreement with data collected in HeLa cells (Park et al., 2003), keratinocytes (Sur et al., 2008) and skeletal muscle (Dodd et al., 2009). However, in U937 human leukemic cells, MEF cells, macrophages (Salari et al., 2013) and rat colon carcinoma REG cells, HSPB1 appears to enhance NF-κB activation in response to either etoposide or TNF-α treatment (Parcellier et al., 2003), suggesting that the outcome of NF-κB regulation by HSPB1 may vary with cell type or stimuli.

CMA, one of the lysosomal proteolysis pathways, is characterized by its specificity for the selective degradation of substrate proteins. Basal levels of CMA activity are detectable in almost all mammalian cells, and contribute to the maintenance of cellular homeostasis as well as specialized functions depending on the cell type and degraded substrate. In contrast to the idea that CMA is upregulated under various conditions ranging from prolonged starvation to different cellular stresses (Cuervo et al., 1995; Dohi et al., 2012), our analysis revealed that the core components of CMA effectors, LAMP-2A, as well as LC3, a marker of macroautophagy, were markedly decreased in NCM-treated microglia. More importantly, only LAMP-2A expression was reversed following Ac2-26 treatment, suggesting that Ac2-26 improves the CMA system in microglia without affecting the macroautophagic pathway. The present study provided the first description of CMA in microglia. Proteins undergoing CMA-mediated lysosomal degradation often contain a loosely defined KFERQ motif, which is important for Hsc70 binding and interactions with LAMP-2A. In this report, we found that both ANXA1 and HSPB1 are CMA substrates. ANXA1 is selectively degraded by CMA (Cuervo et al., 2000). ANXA1 and HSPB1 act as small molecular chaperones associated with IKKβ, which is degraded by CMA. These findings raised the intriguing possibility that ANXA1 or HSPB1 may join with the chaperone Hsc70 and bind to CMA substrates or function independently in facilitating the transport of substrates into lysosomes without interacting with Hsc70, as in canonical CMA. Therefore, the exact roles ofANXA1 and HSPB1 deserve further research.

## Conclusion

In conclusion, our data described a novel mechanism by which exogenous ANXA1 regulates TNF-α expression and provided the first evidence that CMA plays a fundamental role in driving Ac2-26-induced rapid loss of IKKβ protein in microglia. Mechanistically, ANXA1 attenuates IKKβ activity by inducing HSPB1, a small chaperone, to bind to both ANXA1 and IKKβ for degradation in lysosomes. This study presented the first observation that HSPB1 is involved in Ac2-26-induced CMA in microglia. Hence, these results highlighted the important role of CMA in controlling inflammation in microglia and provided a promising therapeutic target pathway applicable to exogenous Ac2-26 in stroke.

## Availability of Data and Material

The datasets used and/or analyzed during the current study are available from the corresponding author on reasonable request.

## Author Contributions

LL designed the experimental project, carried out most of the experiments and prepared the manuscript. DA and JX participated in the primary microglia culture and performed the HSPB1 analysis in tissues and cell. BS and XL contributed to the interpretation of the results. JS provided oversight for the study including experimental design, data interpretation and manuscript preparation. All authors read and approved the final manuscript.

## Conflict of Interest Statement

The authors declare that the research was conducted in the absence of any commercial or financial relationships that could be construed as a potential conflict of interest. The reviewer LP declared a shared affiliation, though no other collaboration with the authors to the handling Editor.

## Supplementary Material

FIGURE S1 Interaction between N-terminal domain of ANXA1 and HSPB1. HEK293T cells were transfected with indicated plasmids for 48 h. CO-IP analysis and immunoblots were performed with indicated antibodies. **(A)** GFP-ANXA1-N interacted with endogenous HSPB1 in HEK293T cells. **(B)** Flag-Ac2-26 interacted with endogenous HSPB1 in HEK293T cells. Data are representative of three independent experiments.

## Abbreviations

ANXA1: Annexin A1
FPR: formyl peptide receptor
OGD/R: oxygen glucose deprivation/reperfusion
NCM: neuronal conditioned medium
LAMP-2A: lysosomal-associated membrane protein 2A
ROS: reactive oxygen species
iNOS: inducible nitric oxide synthase
CMA: chaperone-mediated autophagy.
